# A novel perspective on the selection of an effective approach to reduce road traffic accidents under Fermatean fuzzy settings

**DOI:** 10.1371/journal.pone.0303139

**Published:** 2024-05-10

**Authors:** Dilshad Alghazzawi, Aqsa Noor, Hanan Alolaiyan, Hamiden Abd El-Wahed Khalifa, Alhanouf Alburaikan, Qin Xin, Abdul Razaq

**Affiliations:** 1 Department of Mathematics, College of Science & Arts, King Abdul Aziz University, Rabigh, Saudi Arabia; 2 Department of Mathematics, Division of Science and Technology, University of Education, Lahore, Pakistan; 3 Department of Mathematics, College of Science, King Saud University, Riyadh, Saudi Arabia; 4 Department of Mathematics, College of Science, Qassim University, Buraydah, Saudi Arabia; 5 Faculty of Graduate Studies for Statistical Research, Department of Operations and Management Research, Cairo University, Giza, Egypt; 6 Faculty of Science and Technology, University of the Faroe Islands, Torshavn, Faroe Islands, Denmark; Korea National University of Transportation, REPUBLIC OF KOREA

## Abstract

Road traffic accidents (RTAs) pose a significant hazard to the security of the general public, especially in developing nations. A daily average of more than three thousand fatalities is recorded worldwide, rating it as the second most prevalent cause of death among people aged 5–29. Precise and reliable decisionmaking techniques are essential for identifying the most effective approach to mitigate road traffic incidents. This research endeavors to investigate this specific concern. The Fermatean fuzzy set (FFS) is a strong and efficient method for addressing ambiguity, particularly when the concept of Pythagorean fuzzy set fails to provide a solution. This research presents two innovative aggregation operators: the Fermatean fuzzy ordered weighted averaging (FFOWA) operator and the Fermatean fuzzy dynamic ordered weighted geometric (FFOWG) operator. The salient characteristics of these operators are discussed and important exceptional scenarios are thoroughly delineated. Furthermore, by implementing the suggested operators, we develop a systematic approach to handle multiple attribute decisionmaking (MADM) scenarios that involve Fermatean fuzzy (FF) data. In order to show the viability of the developed method, we provide a numerical illustration encompassing the determination of the most effective approach to alleviate road traffic accidents. Lastly, we conduct a comparative evaluation of the proposed approach in relation to a number of established methodologies.

## 1. Introduction

Multi-attribute decision-making (MADM) problems manifest when a predetermined set of attributes is employed to select one option, action, or nomination from among numerous alternatives. The MADM technique, which is a subfield of operations research and decision science, evaluates complex situations with numerous, and at times, inconsistent, factors. Utilizing the MADM method to manage complex decisions with multiple objectives and trade-offs is beneficial. It aids decision-makers in formulating informed and purposeful judgments that take into account all significant factors, thereby potentially enhancing the outcomes of decisions. MADM is simplified by aggregation operators, which renders it a practical instrument for tackling pragmatic challenges associated with universally prevalent concerns. Aggregation operators seek to merge every separate value into a single value. All values are therefore accounted for in the final aggregate result. Before the discovery of aggregation operators, crisp sets were extensively used as decision-making procedures.

The presence of ambiguity or inadequate information in numerous domains presents significant challenges. Similarly, it can be asserted that decision-making problems often suffer from inadequate and ambiguous information. The resolution of decision-making problems is heavily contingent upon imprecision, uncertainty, and incomplete information. The use of real numbers is inadequate for resolving situations characterized by uncertainty. Thus, fuzzy sets (FSs) provide the resolution in a situation of uncertainty. Zadeh [1] introduced FS in 1965. Specifically, determining the degree of membership of a value within a fuzzy set aids in resolving situations characterized by uncertainty. FSs have advanced significantly in several domains of engineering and technology. Nevertheless, the constituents of traditional FSs are construed solely based on the extent of membership. Uncertainty or partial information in a dataset can be represented by a single membership function. Atanassov [2] defines the extension of the traditional FS idea. The term used to refer to this structure is intuitionistic fuzzy set (IFS).

IFS is constituted by a membership degree and a non-membership degree that must meet the requirement that their sum is equal to or less than 1. Thus, it may effectively convey the ambiguous nature of facts in a more complete and precise manner. The primary challenge that emerges in decision-making challenges is the integration of disparate pieces of information provided by numerous sources in order to reach a judgment or draws inferences. To achieve a high-quality aggregation, researchers employed many methodologies including the utilization of rules, fusion-specific approaches, probability, possibility, and fuzzy set theory. Each of these techniques is based on certain quantitative aggregation operations. These operators are mathematical tools that play a crucial role in reducing a set of values to a single unique value. Various aggregation operations have been developed to combine IF information from different experts, alternatives, and time periods. Various intuitionistic fuzzy aggregation operators were devised and used to solve MADM issues in [3,4]. Li [5] investigated the use of generalized ordered weighted averaging operations to Intuitionistic fuzzy data. Wei [6] proposed the notion of induced geometric aggregation operators in the context of IF information. Since its inception, IFS has received considerable attention and has been utilized effectively to resolve MADM issues [7–9]. However, numerous instances remain in which IFS has been unable to address the problem.

The Pythagorean fuzzy set (PFS) was introduced by Yager [10,11] as a robust extension of the IFS. The sum of the squares of PFS membership and non-membership degrees is inside the interval [0,1]. In comparison to IFS, PFS can handle more uncertain conditions. Therefore, PFS is superior to IFS in terms of its efficacy in solving practical problems. PFSs have quickly garnered the interest of several researchers [12–15]. Pythagorean fuzzy weighted geometric/averaging operators was given by [16]. Zhang [17] introduced the Pythagorean fuzzy ordered weighted averaging (PFOWA) operator. Garg [18] introduced many Einstein operators, applied to Pythagorean fuzzy MADM problems for efficiency. Rahman et al. [19] worked on MADM problems using the Pythagorean fuzzy ordered weighted geometric (PFOWG) operator and its fundamental features. Undoubtedly, the PFS surpasses the IFS in its ability to accurately depict and analyze intricate ambiguity in practical decision-making scenarios. Although PFS offer a wider range of possibilities, it is necessary to create a more sophisticated iteration of fuzzy sets to handle scenarios that are outside the confines of the PFS framework.

Senapati and Yager [20] extends the concepts of IFS and PFS theories and introduced FFS theory. It adheres to the criterion 0 ≤ *μ*^3^ + *ν*^3^ ≤ 1 and offers enhanced adaptability in resolving decision-making situations that involve ambiguity. IFSs are capable of handling a greater degree of uncertainty in MADM problems by providing information about both the degrees of membership and non-membership of the available alternatives. In addition to these benefits, this model had several constraints, such as the constraint that the sum of the membership and non-membership degrees, is limited to 1. PFS is an extension of IFS, distinguished by the condition that the square sum of its membership and non-membership degrees, must not exceed 1. However, in MADM situations, we may encounter a situation where the sum of the degrees of membership and non-membership of a specific attribute exceed 1. For example, if membership degree of an element in a set is 0.9 and non-membership degree is 0.6, then IFS and PFS criteria are not met, due to the sum exceeding 1. In contrast, FFS effectively handles this scenario, by the condition that the cubic sum of its degrees of membership and non-membership does not exceed 1,0.9^3^ + 0.6^3^ = 0.94 ≤ 1. This example illustrates that FFS is more flexible and regarded as a superior tool compared to IFS and PFS in MADM problems. Currently, FFS may be considered the most widespread collection of fuzzy sets. Senapati and Yager [20] presented a formal description of fundamental operations on FFSs and introduced score and accuracy functions for FFSs. In [21], the same authors introduced a new set of operations for FFSs, including subtraction, division, and Fermatean arithmetic mean operations. In the same paper, they also proposed using the Fermatean fuzzy weighted product model to address MCDM problems. In [22], Fermatean fuzzy weighted averaging and geometric operators were defined, along with the investigation of their useful applications in the decision-making domain. FFSs have rapidly captured the attention of many scholars. FF is widely used in MADM problems. The efficacy of employing FF aggregating processes within the COVID-19 testing facility is demonstrated in [23]. The study done in [24] analyzed many scoring functions for FFS and assessed their practical applicability in the domain of transportation issues and decision-making. The concept for the identification of an effective sanitizer to limit the transmission of COVID-19 under FF envirnoment is presented in [25]. The weighted aggregated sum product assessment approach was developed in [26] within the context of the FF environment. Many Fermatean fuzzy capital budgeting approaches have been presented. In [27]. A recent work [28] has offered a comprehensive approach for determining the most effective treatment methods for blood cancer in a Fermatean fuzzy setting. In [29] a consensus-based process for selecting healthcare waste treatment system using FF knowledge is proposed. A new notion of complex Fermatean neutrosophic graph was introduced in [30]. Several scholars have extensively studied the intricate structure of FFSs throughout several fields. For example, interval-valued FF Dombi aggregation operators [31], interval-valued FF TOPSIS approach and its relevance to the sustainability system [32], q-rung orthopair fuzzy Frank aggregation operators and its application in MADM [33], multiple attribute group decision making based on quasirung orthopair fuzzy sets [34], MADM based on quasirung fuzzy sets [35] and some picture fuzzy aggregation operators based on Frank t-norm and t-conorm [36].

The exploration of aggregation operators is a captivating field of study in the domain of decision making. In the last few decades, several operators have been suggested, including the ordered weighted averaging (OWA) and ordered weighted geometric (OWG) operators. Ordered weighted aggregation operators Ordered weighted aggregation operators possess great ability to handle imprecise information. Yager [37] introduced the OWA operator, which has been extensively applied in resolving numerous problems. This concept has been examined both theoretically [38–40] and from an implicational perspective. This method has also been utilized in linguistic decision-making assessments [41], eliminating noise in computer vision [42], offering a breakdown of all rank-dependent poverty measures in terms of inequality, intensity, and incidence [43], and enabling experts to express multiple levels of self-confidence when stating their inclinations [44]. The introduction of OWG operator by Chiclana [45] incorporates the notion of fuzzy majority in decision-making procedures with ratio-scale evaluations, comparable to the OWA operator [46]. OWG utilizes the OWA operator and the geometric mean. The comprehensive examination of the genesis and applications of the OWG method in MADM can be found in reference [47].

### 1.1. Research gap

We can see from references [3,4,17,19] that the theories developed for ordered weighted aggregation operators are based on IFSs and PFSs. Thus, there are many MADM problems, that IFS and PFS environment cannot handle because the sum of degrees of membership and non-membership and the square sum of degrees of membership and non-membership exceed 1. As a result, when decision-makers come up the situations like (0.9, 0.6), or (0.8, 0.7) then the notions developed in references [3,4,17,19] fail to tackle such kind of data. It means that, there is need to focus on enhancing proficiency in some advanced structural concepts. Moreover, weighted aggregation operators involve assigning predetermined weights to different attributes and lack versatility, making them less suitable for handling uncertainty. The possibility of information loss is a concern because low-weighted data may have the smallest effect on the overall aggregation, leading to an insufficient depiction of the data. One can observe these limitations in [3,4,16,22]. Thus, there is a need to develop notions based on ordered weighted aggregation operator on some advance structure to overcome these issues.

### 1.2. Motivation

FFSs accommodate greater degrees of uncertainty than IFSs and PFSs, highlighting their suitability for managing situations characterized by increased ambiguity and complexity in MADM problems. For instant, these sets easily handles scenario like (0.9, 0.6), or (0.8, 0.7), by the condition that the cubic sum of their degrees of membership and non-membership does not exceed 1. Ordered weighted aggregation operators do not reliant on predetermined weights allocated to specific attribute. These operators enable decision-makers to include the uncertainty and imprecision of real-world situations by introducing reordering of the input values that precisely convey their significance in the decision-making procedure. The ability of ordered weighted aggregation operators to be flexible is especially beneficial when handling subjective and ambiguous data, since it allows decision-makers to present their preferences without being confined by specific weight values. Although these operators have been established for classical fuzzy, intuitionistic fuzzy, and Pythagorean fuzzy environments, there is a dearth of research discussing their application to data involving FFSs. In order to rectify this deficiency, it is critical to establish concrete operators for FFSs that can efficiently manage such settings.

The primary contributions of this work are delineated as follows:

- Two innovative aggregation operators, FFOWA and FFOWG operators, are defined to deal with complex decision-making scenarios involving FF data.
- The structural characteristics of the proposed operators are proved. This highlights the logical existence of these operators.
- With the help of newly designed operators, a rigorous approach to solve MADM problems in the framework of FF knowledge is provided.
- The suggested approach is demonstrated by applying it to the resolution of a practical MADM issue, such as determining the best course of action to reduce traffic accidents.
- A comprehensive comparative analysis is undertaken to evaluate the feasibility of the proposed approach in comparison to several existing techniques.

This manuscript’s succeeding sections are organized as follows: Section 2 provides essential terminology for understanding this manuscript’s main discoveries. In the third section, ordered weighted aggregate operators for FFS are introduced, and their essential features are examined. Section 4 develops a step-by-step mathematical technique to solve MADM issues utilizing FF information and ordered weighted aggregating operators. The purpose of Section 5 is to demonstrate how the proposed method can be utilized to determine the most effective strategy for reducing RTAs. In addition, a comparison study is undertaken to evaluate the viability and efficacy of this novel approach relative to traditional approaches. The conclusion of this research is outlined in Section 6.

The specifics of the symbols and abbreviations are provided in Tables 1 and 2, respectively.

**Table 1:** List of abbreviations.

Abbreviations	Explanation	Abbreviations	Explanation
MADM	Multi-attribute decision-making	PFS	Pythagorean fuzzy set
FS	Fuzzy set	OWA	Ordered weighted averaging
FF	Fermatean fuzzy	OWG	Ordered weighted geometric
FFS	Fermatean fuzzy set	FFOWA	Fermatean fuzzy ordered weighted averaging
FFN	Fermatean fuzzy Number	FFOWG	Fermatean fuzzy ordered weighted geometric
IFS	Intuitionistic fuzzy set	RTAs	Road traffic accidents

**Table 2:** List of symbols.

Notation	Description	Notation	Description
*Ψ*	Universe	S	Score function
*Ω*	Fermatean fuzzy set	H	Accuracy function
*ξ*	Element of universe	R	Decision matrix
*μ*	Membership function	$R_{\sigma }$	Permuted decision matrix
*ν*	Non-membership function	*ϒ*	Alternative
*ω*	Weight vector	*χ*	Attribute

## 2. Preliminaries

This section contains the essential descriptions of the terminology required to comprehend the main findings of this article.

**Definition 1**. [20]. In the context of a universe of discourse *Ψ*, a FFS, *Ω*, is defined as follows:

$$\Omega =\left\{\xi ,\;\mu_{\Omega }\left(\xi \right),\;\nu_{\Omega }\left(\xi \right):\xi \in \Psi \right\},$$

where, *μ*_*Ω*_∶ *Ψ* → [0,1] describes membership function and *ν*_*Ω*_∶ *Ψ* → [0,1] describes non-membership function satisfying $0\leq \mu_{\Omega }^{3}(\xi )+\nu_{\Omega }^{3}(\xi )\leq 1\;\forall \xi \in \Psi$.

Moreover, for any element *ξ* ∈ *Ψ*, the indeterminacy degree of *ξ* in context of *Ω*, is described as $\pi_{\Omega }\left(\xi \right)=\sqrt[{3}]{1-\mu_{\Omega }^{3}\left(\xi \right)-\nu_{\Omega }^{3}(\xi )}$.

Furthermore, we express the degrees of membership and non-membership of *ξ* in *Ψ* as *ξ* = (*μ*_*Ω*_, *ν*_*Ω*_), which is referred to as a Fermatean fuzzy number (FFN). Here, *μ*_*Ω*_, *ν*_*Ω*_ ∈ [0,1] and satisfy the condition $0\leq \mu_{\Omega }^{3}+\nu_{\Omega }^{3}\leq 1$.

**Definition 2**. [20]. Consider two FFNs, $\Omega_{1}=\left(\mu_{\Omega_{1}},\;\nu_{\Omega_{1}}\right)$ and $\Omega_{2}=\left(\mu_{\Omega_{2}},\;\nu_{\Omega_{2}}\right)$. The fundamental operational laws that regulate their interrelations are as follows:

- *Ω*_1_ ≤ *Ω*_2_, if $\mu_{\Omega_{1}}\leq \mu_{\Omega_{2}}$ and $\nu_{\Omega_{1}}\leq \nu_{\Omega_{2}}$
- *Ω*_1_ = *Ω*_2_ if and only if *Ω*_1_ ⊆ *Ω*_2_ and *Ω*_2_ ⊆ *Ω*_1_
-
$\Omega_{1}^{c}=\left(\nu_{\Omega_{1}},\mu_{\Omega_{1}}\right)$

**Definition 3**. [20]. Let $\Omega =\left(\mu_{\Omega },\;\nu_{\Omega }\right),\;\Omega_{1}=\left(\mu_{\Omega_{1}},\;\nu_{\Omega_{1}}\right)$ and $\Omega_{2}=\left(\mu_{\Omega_{2}},\;\nu_{\Omega_{2}}\right)$ be three FFNs and *ω* > 0, then

-
$\Omega_{1}⊕\Omega_{2}=\left(\sqrt[{3}]{\mu_{\Omega_{1}}^{3}+\mu_{\Omega_{2}}^{3}-\mu_{\Omega_{1}}^{3}\mu_{\Omega_{2}}^{3}},\;\nu_{\Omega_{1}}\nu_{\Omega_{2}}\right)$

-
$\Omega_{1}⊗\Omega_{2}=\left(\mu_{\Omega_{1}}\mu_{\Omega_{2}},\sqrt[{3}]{\nu_{\Omega_{1}}^{3}+\nu_{\Omega_{2}}^{3}-\nu_{\Omega_{1}}^{3}\nu_{\Omega_{2}}^{3}}\;\right)$

-
$\omega \Omega =\left(\sqrt[{3}]{1-{\left(1-\mu_{\Omega }^{3}\right)}^{\omega }},\;\nu_{\Omega }^{\omega }\right)$

-
$\Omega^{\omega }=\left(\mu_{\Omega }^{\omega },\;\sqrt[{3}]{1-{\left(1-\nu_{\Omega }^{3}\right)}^{\omega }}\;\right)$

**Definition 4**. [20]. In the following, we define two crucial functions for every FFN *Ω* = (*μ*_*Ω*_, *ν*_*Ω*_):

- The expression for the score function S(Ω) is $\mu_{\Omega }^{3}-\nu_{\Omega }^{3}$. The result in this case falls in [−1, 1].
- The expression for the score function H(Ω) is $\mu_{\Omega }^{3}+\nu_{\Omega }^{3}$. The result in this case falls in [0, 1].

Also, *Ω*_1_ and *Ω*_2_ fulfill the subsequent comparison rules:

- $S\left(\Omega_{1}\right)≻S\left(\Omega_{2}\right)$ implies *Ω*_1_ ≻ *Ω*_2_
- $S\left(\Omega_{1}\right)≺S\left(\Omega_{2}\right)$ implies *Ω*_1_ ≺ *Ω*_2_
- If $S\left(\Omega_{1}\right)=S\left(\Omega_{2}\right)$, then $H\left(\Omega_{1}\right)≻H\left(\Omega_{2}\right)$ implies $\Omega_{1}≻\Omega_{2},\;H\left(\Omega_{1}\right)≺H\left(\Omega_{2}\right)$ implies *Ω*_1_ ≺ *Ω*_2_ and $H\left(\Omega_{1}\right)=H\left(\Omega_{2}\right)$ implies *Ω*_1_ ~ *Ω*_2_

## 3. Fundamental properties of FFOW aggregation operators

This section provides an introduction to the FFOWA operator and FFOWG operator, and explores their essential properties.

**Definition 5**. Let *i* = 1,2, …, *n* and $\Omega_{i}=(\mu_{\Omega_{i}},\;\nu_{\Omega_{i}})$ be a collection F of FFNs. Suppose that *ω* = (*ω*_1_, *ω*_2_, …, *ω*_*n*_)^*T*^ is the associated weight vector of *Ω*_*i*_ with *ω*_*i*_ ∈ [0,1] and $\sum_{i=1}^{n}\omega_{i}=1$. Then FFOWA operator is a function $FFOWA:F^{n}\to F$, where

$$FFOWA\left(\Omega_{1},\Omega_{2},\ldots ,\Omega_{n}\right)={⊕}_{i=1}^{n}\omega_{i}.\Omega_{\sigma \left(i\right)}$$

$$FFOWA\left(\Omega_{1},\Omega_{2},\ldots ,\Omega_{n}\right)=\left(\sqrt[{3}]{1-\prod_{i=1}^{n}{\left(1-\mu_{\Omega_{\sigma \left(i\right)}}^{3}\right)}^{\omega_{i}}},\;\prod_{i=1}^{n}\nu_{\Omega_{\sigma \left(i\right)}}^{\omega_{i}}\right)$$

where (*σ*(1), *σ*(2), …, *σ*(*n*)) is the permutation of *i* = 1,2,3, …, *n*, such that *Ω*_*σ*(*i*−1)_ ≥ *Ω*_*σ*(*i*)_, for all *i*.

**Theorem 1**. Let *i* = 1,2, …, *n* and $\Omega_{i}=(\mu_{\Omega_{i}},\nu_{\Omega_{i}})$ denote FFNs. The outcome of aggregating these FFNs via the FFOWA operator is maintained as an FFN. The expression for it is as follows:

$$FFOWA\left(\Omega_{1},\Omega_{2},\ldots ,\Omega_{n}\right)=\left(\sqrt[{3}]{1-\prod_{i=1}^{n}{\left(1-\mu_{\Omega_{\sigma \left(i\right)}}^{3}\right)}^{\omega_{i}}},\;\prod_{i=1}^{n}\nu_{\Omega_{\sigma \left(i\right)}}^{\omega_{i}}\right)$$

where, *ω* = (*ω*_1_, *ω*_2_, …, *ω*_*n*_)^T^ be the associated weight vector of *Ω*_*i*_ with some conditions *ω*_*i*_ ∈ [0,1] and $\sum_{i=1}^{n}\omega_{i}=1$.

**Proof**. To prove this theorem, we use mathematical induction on *n*. If *n* = 2, then

$$FFOWA\left(\Omega_{1},\Omega_{2}\right)=\omega_{1}.\Omega_{\sigma \left(1\right)}⊕\omega_{2}.\Omega_{\sigma \left(2\right)}$$

Breaking down the components *ω*_1_.*Ω*_*σ*(1)_ and *ω*_2_.*Ω*_*σ*(2)_, in view of Definition 5, we obtain

$$\begin{array}{l} \omega_{1}.\Omega_{\sigma \left(1\right)}=\left(\;\sqrt[{3}]{1-{\left(1-\mu_{\Omega_{\sigma \left(1\right)}}^{3}\right)}^{\omega_{1}}},\;\nu_{\Omega_{\sigma \left(1\right)}}^{\omega_{1}}\right)\\ \omega_{2}.\;\Omega_{\sigma \left(2\right)}=\left(\;\sqrt[{3}]{1-{\left(1-\mu_{\Omega_{\sigma \left(2\right)}}^{3}\right)}^{\omega_{2}}},\;\nu_{\Omega_{\sigma \left(2\right)}}^{\omega_{2}}\right) \end{array}$$

Then,

$$\begin{array}{l} \omega_{1}.\;\Omega_{\sigma \left(1\right)}⊕\omega_{2}.\;\Omega_{\sigma \left(2\right)}\\ \;=\left(\sqrt[{3}]{1-{\left(1-\mu_{\Omega_{\sigma \left(1\right)}}^{3}\right)}^{\omega_{1}}},\;\nu_{\Omega_{\sigma \left(1\right)}}^{\omega_{1}}\right)⊕\left(\;\sqrt[{3}]{1-{\left(1-\mu_{\Omega_{\sigma \left(2\right)}}^{3}\right)}^{\omega_{2}}},\;\nu_{\Omega_{\sigma \left(2\right)}}^{\omega_{2}}\right)\\ \;=\left(\sqrt[{3}]{1-{\left(1-\mu_{\Omega_{\sigma \left(1\right)}}^{3}\right)}^{\omega_{1}}{\left(1-\mu_{\Omega_{\sigma \left(2\right)}}^{3}\right)}^{\omega_{2}}},\;\nu_{\Omega_{\sigma \left(1\right)}}^{\omega_{1}}\nu_{\Omega_{\sigma \left(2\right)}}^{\omega_{2}}\right) \end{array}$$

Consequently,

$$FFOWA\left(\Omega_{1},\Omega_{2}\right)=\left(\sqrt[{3}]{1-\prod_{i=1}^{2}{\left(1-\mu_{\Omega_{\sigma \left(i\right)}}^{3}\right)}^{\omega_{i}}},\;\prod_{i=1}^{2}\nu_{\Omega_{\sigma \left(i\right)}}^{\omega_{i}}\right)$$

This means that the theorem works for *n* = 2.

Then, assuming the theorem is valid for *n* = *k* > 2, we obtain:

$$FFOWA\left(\Omega_{1},\Omega_{2},\ldots ,\Omega_{k}\right)={⊕}_{i=1}^{k}\omega_{i}\Omega_{\sigma \left(i\right)}$$

$$FFOWA\left(\Omega_{1},\Omega_{2},\ldots ,\Omega_{k}\right)=\left(\sqrt[{3}]{1-\prod_{i=1}^{k}{\left(1-\mu_{\Omega_{\sigma \left(i\right)}}^{3}\right)}^{\omega_{i}}},\;\prod_{i=1}^{k}\nu_{\Omega_{\sigma \left(i\right)}}^{\omega_{i}}\right)$$

Now, for the case *n* = *k* + 1, we can evaluate it as:

$$\begin{array}{l} FFOWA\left(\Omega_{1},\Omega_{2},\ldots ,\Omega_{k},\Omega_{k+1}\right)={⊕}_{i=1}^{k}\omega_{i}\Omega_{\sigma \left(i\right)}⊕\omega_{k+1}.\Omega_{\sigma \left(k+1\right)}\\ \;=\left(\sqrt[{3}]{1-\prod_{i=1}^{k}{\left(1-\mu_{\Omega_{\sigma \left(i\right)}}^{3}\right)}^{\omega_{i}}},\;\prod_{i=1}^{k}\nu_{\Omega_{\sigma \left(i\right)}}^{\omega_{i}}\right)\\ \;⊕\left(\sqrt[{3}]{1-{\left(1-\mu_{\Omega_{\sigma \left(k+1\right)}}^{3}\right)}^{\omega_{k+1}}},\;\nu_{\Omega_{\sigma \left(k+1\right)}}^{\omega_{k+1}}\right) \end{array}$$

This mean that

$$FFOWA\left(\Omega_{1},\Omega_{2},\ldots ,\Omega_{k+1}\right)=\left(\sqrt[{3}]{1-\prod_{i=1}^{k+1}{\left(1-\mu_{\Omega_{\sigma \left(i\right)}}^{3}\right)}^{\omega_{i}}},\;\prod_{i=1}^{k+1}\nu_{\Omega_{\sigma \left(i\right)}}^{\omega_{i}}\right)$$

This proves that the theorem remains valid when *n* equals *k* + 1. Thus, it can be deduced that the assertion holds true for every value of *n*.

The following example shows the application of Theorem 1.

**Example 1**. Let *Ω*_1_ = (0.7, 0.6), *Ω*_2_ = (0.8, 0.4), *Ω*_3_ = (0.9, 0.5) and *Ω*_4_ = (0.8, 0.7) be four FFNs and *ω* = (0.1, 0.2, 0.3, 0.4)^*T*^ be the associated weight vector of *Ω*_*i*_. First we calculate the scores of *Ω*_*i*_ by means of Definition 4,

$$S\left(\Omega_{1}\right)=0.127,S\left(\Omega_{2}\right)=0.448,S\left(\Omega_{3}\right)=0.604,S\left(\Omega_{4}\right)=0.169$$

Since $S\left(\Omega_{3}\right)≻S\left(\Omega_{2}\right)≻S\left(\Omega_{4}\right)≻S\left(\Omega_{1}\right)$, then the permutation vector *Ω*_*σ*(*i*)_, where *i* = 1,2,3,4, is described as follows:

$$\left(\Omega_{\sigma \left(1\right)},\Omega_{\sigma \left(2\right)},\Omega_{\sigma \left(3\right)},\Omega_{\sigma \left(4\right)}\right)=\left(\left(0.9,0.5\right),\left(0.8,0.4\right),\left(0.8,0.7\right),\left(0.7,0.6\right)\right)$$

Thus, considering Definition 5, the following result is obtained:

$$FFOWA\left(\Omega_{1},\Omega_{2},\;\Omega_{3},\Omega_{4}\right)=\left(0.783,0.568\right)$$

**Theorem 2**. (Idempotency) Let *i* = 1,2, …, *n* and $\Omega_{i}=(\mu_{\Omega_{i}},\nu_{\Omega_{i}})$ denote FFNs. Suppose that *ω* = (*ω*_1_, *ω*_2_, …, *ω*_*n*_)^T^ is the associated weight vector of *Ω*_*i*_ such that *ω*_*i*_ ∈ [0,1] and $\sum_{i=1}^{n}\omega_{i}=1$. If *Ω*_*σ*(*i*)_ = *Ω*_*σ*(*j*)_ are mathematically identical where $\Omega_{\sigma (j)}=\left(\mu_{\Omega_{\sigma (j)}},\nu_{\Omega_{\sigma (j)}}\right)$ then

$$FFOWA\left(\Omega_{1},\Omega_{2},\ldots ,\Omega_{n}\right)=\Omega_{\sigma \left(j\right)}.$$

**Proof**. Given that *Ω*_*σ*(*i*)_ = *Ω*_*σ*(*j*)_, for some *j* ∈ {1,2, …, *n*} implying $\mu_{\Omega_{\sigma (i)}}=\mu_{\Omega_{\sigma (j)}}$ and $\nu_{\Omega_{\sigma (i)}}=\nu_{\Omega_{\sigma (j)}}$, then by using the above fact in Definition 5, we get

$$\begin{array}{ll} FFOWA\left(\Omega_{1},\Omega_{2},\ldots ,\Omega_{n}\right) & =\left(\sqrt[{3}]{1-\prod_{i=1}^{n}{\left(1-\mu_{\Omega_{\sigma \left(i\right)}}^{3}\right)}^{\omega_{i}}},\;\prod_{i=1}^{n}\nu_{\Omega_{\sigma \left(i\right)}}^{\omega_{i}}\right)\\ & =\left(\sqrt[{3}]{1-{\left(1-\mu_{\Omega_{\sigma \left(j\right)}}^{3}\right)}^{\sum_{i=1}^{n}\omega_{i}}},\;\nu_{\Omega_{\sigma \left(j\right)}}^{\sum_{i=1}^{n}\omega_{i}}\right)\\ & =\left(\sqrt[{3}]{1-\left(1-\mu_{\Omega_{\sigma \left(j\right)}}^{3}\right)},\;\nu_{\Omega_{\sigma \left(j\right)}}\right)=\left(\sqrt[{3}]{\mu_{\Omega_{\sigma \left(j\right)}}^{3}},\;\nu_{\Omega_{\sigma \left(j\right)}}\right)=\left(\mu_{\Omega_{\sigma \left(j\right)}},\nu_{\Omega_{\sigma \left(j\right)}}\right) \end{array}$$

Consequently,

$$FFOWA\left(\Omega_{1},\Omega_{2},\ldots ,\Omega_{n}\right)=\Omega_{\sigma \left(j\right)}$$

**Theorem 3**. (Boundedness) Let *i* = 1,2, …, *n* and $\Omega_{i}=(\mu_{\Omega_{i}},\nu_{\Omega_{i}})$ denote FFNs. Suppose that $\Omega^{-}=\left({\text{min}}_{i}\left\{\mu_{\Omega_{\sigma (i)}}\right\},{\text{max}}_{i}\left\{\nu_{\Omega_{\sigma (i)}}\right\}\right)$ and $\Omega^{+}=\left({\text{max}}_{i}\left\{\mu_{\Omega_{\sigma (i)}}\right\},{\text{min}}_{i}\left\{\nu_{\Omega_{\sigma (i)}}\right\}\right)$ are the lower and upper bounds of $\Omega_{i}=\left(\mu_{\Omega_{i}},\;\nu_{\Omega_{i}}\right)$. Moreover, *ω* = (*ω*_1_, *ω*_2_, …, *ω*_*n*_)^T^ is the associated weight vector of *Ω*_*i*_ satisfying the conditions *ω*_*i*_ ∈ [0,1] and $\sum_{i=1}^{n}\omega_{i}=1$. Then

$$\Omega^{-}\leq FFOWA\left(\Omega_{1},\;\Omega_{2},\ldots ,\;\Omega_{n}\right)\leq \Omega^{+}.$$

**Proof**. Let us apply FFOWA operator on the set of FFNs, as follows:

$$FFOWA\left(\Omega_{1},\Omega_{2},\ldots ,\Omega_{n}\right)=\left(\mu_{\Omega },\;\nu_{\Omega }\right),$$

where *Ω* = (*μ*_*Ω*_, *ν*_*Ω*_). For each $\mu_{\Omega_{\sigma (i)}}$, we have

$$\begin{array}{c} \underset{i}{\text{min}}\left\{\mu_{\Omega_{\sigma \left(i\right)}}\right\}\leq \mu_{\Omega_{\sigma \left(i\right)}}\leq \underset{i}{\text{max}}\left\{\mu_{\Omega_{\sigma \left(i\right)}}\right\}\\ \Rightarrow \underset{i}{\text{min}}\left\{\mu_{\Omega_{\sigma \left(i\right)}}^{3}\right\}\leq \mu_{\Omega_{\sigma \left(i\right)}}^{3}\leq \underset{i}{\text{max}}\left\{\mu_{\Omega_{\sigma \left(i\right)}}^{3}\right\}\\ \Rightarrow 1-\underset{i}{\text{max}}\left\{\mu_{\Omega_{\sigma \left(i\right)}}^{3}\right\}\leq 1-\mu_{\Omega_{\sigma \left(i\right)}}^{3}\leq 1-\underset{i}{\text{min}}\left\{\mu_{\Omega_{\sigma \left(i\right)}}^{3}\right\}\\ \Rightarrow \prod_{i=1}^{n}{\left(1-\underset{i}{\text{max}}\left\{\mu_{\Omega_{\sigma \left(i\right)}}^{3}\right\}\right)}^{\omega_{i}}\leq \prod_{i=1}^{n}{\left(1-\mu_{\Omega_{\sigma \left(i\right)}}^{3}\right)}^{\omega_{i}}\leq \prod_{i=1}^{n}{\left(1-\underset{i}{\text{min}}\left\{\mu_{\Omega_{\sigma \left(i\right)}}^{3}\right\}\right)}^{\omega_{i}}\\ \Rightarrow {\left(1-\underset{i}{\text{max}}\left\{\mu_{\Omega_{\sigma \left(i\right)}}^{3}\right\}\right)}^{\sum_{i=1}^{n}\omega_{i}}\leq \prod_{i=1}^{n}{\left(1-\mu_{\Omega_{\sigma \left(i\right)}}^{3}\right)}^{\omega_{i}}\leq {\left(1-\underset{i}{\text{min}}\left\{\mu_{\Omega_{\sigma \left(i\right)}}^{3}\right\}\right)}^{\sum_{i=1}^{n}\omega_{i}}\\ \Rightarrow \left(1-\underset{i}{\text{max}}\left\{\mu_{\Omega_{\sigma \left(i\right)}}^{3}\right\}\right)\leq \prod_{i=1}^{n}{\left(1-\mu_{\Omega_{\sigma \left(i\right)}}^{3}\right)}^{\omega_{i}}\leq \left(1-\underset{i}{\text{min}}\left\{\mu_{\Omega_{\sigma \left(i\right)}}^{3}\right\}\right)\\ \Rightarrow \underset{i}{\text{min}}\left\{\mu_{\Omega_{\sigma \left(i\right)}}^{3}\right\}\leq 1-\prod_{i=1}^{n}{\left(1-\mu_{\Omega_{\sigma \left(i\right)}}^{3}\right)}^{\omega_{i}}\leq \underset{i}{\text{max}}\left\{\mu_{\Omega_{\sigma \left(i\right)}}^{3}\right\}\\ \Rightarrow \sqrt[{3}]{{\text{min}}_{i}\left\{\mu_{\Omega_{\sigma \left(i\right)}}^{3}\right\}}\leq \sqrt[{3}]{1-\prod_{i=1}^{n}{\left(1-\mu_{\Omega_{\sigma \left(i\right)}}^{3}\right)}^{\omega_{i}}}\leq \sqrt[{3}]{{\text{max}}_{i}\left\{\mu_{\Omega_{\sigma \left(i\right)}}^{3}\right\}}\\ \Rightarrow \underset{i}{\text{min}}\left\{\mu_{\Omega_{\sigma \left(i\right)}}\right\}\leq \mu_{\Omega }\leq \underset{i}{\text{max}}\left\{\mu_{\Omega_{\sigma \left(i\right)}}\right\} \end{array}$$ (1)

Moreover,

$$\begin{array}{c} \underset{i}{\text{min}}\left\{\nu_{\Omega_{\sigma \left(i\right)}}\right\}\leq \nu_{\Omega_{\sigma \left(i\right)}}\leq \underset{i}{\text{max}}\left\{\nu_{\Omega_{\sigma \left(i\right)}}\right\}\\ \Rightarrow \prod_{i=1}^{n}{\left(\underset{i}{\text{min}}\left\{\nu_{\Omega_{\sigma \left(i\right)}}\right\}\right)}^{\omega_{i}}\leq \prod_{i=1}^{n}{\left(\nu_{\Omega_{\sigma \left(i\right)}}\right)}^{\omega_{i}}\leq \prod_{i=1}^{n}{\left(\underset{i}{\text{max}}\left\{\nu_{\Omega_{\sigma \left(i\right)}}\right\}\right)}^{\omega_{i}}\\ \Rightarrow {\left(\underset{i}{\text{min}}\left\{\nu_{\Omega_{\sigma \left(i\right)}}\right\}\right)}^{\sum_{i=1}^{n}\omega_{i}}\leq \prod_{i=1}^{n}{\left(\nu_{\Omega_{\sigma \left(i\right)}}\right)}^{\omega_{i}}\leq {\left(\underset{i}{\text{max}}\left\{\nu_{\Omega_{\sigma \left(i\right)}}\right\}\right)}^{\sum_{i=1}^{n}\omega_{i}}\\ \Rightarrow \underset{i}{\text{min}}\left\{\nu_{\Omega_{\sigma \left(i\right)}}\right\}\leq \nu_{\Omega }\leq \underset{i}{\text{max}}\left\{\nu_{\Omega_{\sigma \left(i\right)}}\right\} \end{array}$$ (2)

Hence, by comparing relations 1 and 2, we obtain that

$$\Omega^{-}\leq FFOWA\left(\Omega_{1},\;\Omega_{2},\ldots ,\;\Omega_{n}\right)\leq \Omega^{+}$$

**Theorem 4**. Consider two collections of FFNs $\Omega_{i}=\left(\mu_{\Omega_{i}},\;\nu_{\Omega_{i}}\right)\;$ and $\Omega_{i}^{\prime }=\left(\mu_{\Omega_{i}^{\prime }},\;\nu_{\Omega_{i}^{\prime }}\right)$, with *i* ranging from 1 to *n* and *ω* = (*ω*_1_, *ω*_2_, …, *ω*_*n*_)^*T*^ be the associated weight vector of *Ω*_*i*_ and $\Omega_{i}^{\prime }$ satisfying the constraints *ω*_*i*_ ∈ [0,1] and $\sum_{i=1}^{n}\omega_{i}=1$. If $\mu_{\Omega_{\sigma (i)}}\leq \mu_{\Omega_{\sigma \left(i\right)}^{\prime }}$ and $\nu_{\Omega_{\sigma (i)}}\geq \nu_{\Omega_{\sigma \left(i\right)}^{\prime }}$, then we can establish that:

$$FFOWA\left(\Omega_{1},\;\Omega_{2},\ldots ,\;\Omega_{n}\right)\leq FFOWA\left(\Omega_{1}^{\prime },\;\Omega_{2}^{\prime },\ldots \;,\;\Omega_{n}^{\prime }\right)$$

**Proof**. The application of FFOWA on *Ω*_*i*_ and $\Omega_{i}^{\prime }$ gives the following:

$$FFDWA\left(\Omega_{1},\;\Omega_{2},\ldots ,\;\Omega_{n}\right)=\left(\mu_{\Omega },\;\nu_{\Omega }\right)\;\text{and}\;FFDWA\left(\Omega_{1}^{\prime },\;\Omega_{2}^{\prime },\;\;\ldots ,\;\Omega_{n}^{\prime }\right)=\left(\mu_{\Omega^{\prime }},\;\nu_{\Omega^{\prime }}\right)$$

Since $\mu_{\Omega_{\sigma (i)}}\leq \mu_{\Omega_{\sigma \left(i\right)}^{'}}$, which implies that $\mu_{\Omega_{\sigma (i)}}^{3}\leq \mu_{\Omega_{\sigma \left(i\right)}^{'}}^{3}$, we can deduce that

$$\begin{array}{c} 1-\mu_{\Omega_{\sigma \left(i\right)}}^{3}\geq 1-\mu_{\Omega_{\sigma \left(i\right)}^{\prime }}^{3}\\ \Rightarrow \prod_{i=1}^{n}{\left(1-\mu_{\Omega_{\sigma \left(i\right)}}^{3}\right)}^{\omega_{i}}\geq \prod_{i=1}^{n}{\left(1-\mu_{\Omega_{\sigma \left(i\right)}^{\prime }}^{3}\right)}^{\omega_{i}}\\ \Rightarrow 1-\prod_{i=1}^{n}{\left(1-\mu_{\Omega_{\sigma \left(i\right)}}^{3}\right)}^{\omega_{i}}\leq 1-\prod_{i=1}^{n}{\left(1-\mu_{\Omega_{\sigma \left(i\right)}^{\prime }}^{3}\right)}^{\omega_{i}}\\ \Rightarrow \sqrt[{3}]{1-\prod_{i=1}^{n}{\left(1-\mu_{\Omega_{\sigma \left(i\right)}}^{3}\right)}^{\omega_{i}}}\leq \sqrt[{3}]{1-\prod_{i=1}^{n}{\left(1-\mu_{\Omega_{\sigma \left(i\right)}^{\prime }}^{3}\right)}^{\omega_{i}}} \end{array}$$

Hence we can conclude that,

$$\mu_{\Omega }\leq \mu_{\Omega^{\prime }}$$ (3)

Similarly, by considering $\nu_{\Omega_{\sigma (i)}}\geq \nu_{\Omega_{\sigma \left(i\right)}^{\prime }}$, we derive:

$$\prod_{i=1}^{n}\nu_{\Omega_{\sigma \left(i\right)}}^{\omega_{i}}\geq \prod_{i=1}^{n}\nu_{\Omega_{\sigma \left(i\right)}^{\prime }}^{\omega_{i}}$$

Which implies,

$$\nu_{\Omega }\geq \nu_{\Omega^{\prime }}$$ (4)

Therefore, by comparing 3 and 4 and utilizing the Definition 5, we established the desire result,

$$FFOWA\left(\Omega_{1},\;\Omega_{2},\ldots ,\;\Omega_{n}\right)\leq FFOWA\left(\Omega_{1}^{\prime },\;\Omega_{2}^{\prime },\ldots ,\;\Omega_{n}^{\prime }\right)$$

In our subsequent definition, we introduce an ordered weighted geometric aggregation operator designed for the FFNs, namely the Fermatean fuzzy ordered weighted geometric (FFOWG) operator. Additionally, we investigate its structural characteristics.

**Definition 6**. Let *i* = 1,2, …, *n* and $\Omega_{i}=(\mu_{\Omega_{i}},\;\nu_{\Omega_{i}})$ be a collection F of FFNs. Suppose that and *ω* = (*ω*_1_, *ω*_2_, …, *ω*_*n*_)^*T*^ is the associated weight vector of *Ω*_*i*_ with *ω*_*i*_ ∈ [0,1] and $\sum_{i=1}^{n}\omega_{i}=1$. Then, FFOWG operator is a mapping $FFOWG:F^{n}\to F$, defined by the following rule:

$$FFOWG\left(\Omega_{1},\Omega_{2},\ldots ,\Omega_{n}\right)={⊗}_{i=1}^{n}\Omega_{\sigma \left(i\right)}^{\omega_{i}}$$

$$=\left(\prod_{i=1}^{n}\mu_{\Omega_{\sigma \left(i\right)}}^{\omega_{i}},\sqrt[{3}]{1-\prod_{i=1}^{n}{\left(1-\nu_{\Omega_{\sigma \left(i\right)}}^{3}\right)}^{\omega_{i}}}\right)$$

**Theorem 5**. Let *i* = 1,2, …, *n* and $\Omega_{i}=(\mu_{\Omega_{i}},\nu_{\Omega_{i}})$ denote FFNs. The outcome of aggregating these FFNs via the FFOWG operator is maintained as an FFN. The expression for it is as follows:

$$FFOWG\left(\Omega_{1},\Omega_{2},\ldots ,\Omega_{n}\right)=\left(\prod_{i=1}^{n}\mu_{\Omega_{\sigma \left(i\right)}}^{\omega_{i}},\;\sqrt[{3}]{1-\prod_{i=1}^{n}{\left(1-\nu_{\Omega_{\sigma \left(i\right)}}^{3}\right)}^{\omega_{i}}}\right)$$

where, *ω* = (*ω*_1_, *ω*_2_, …, *ω*_*n*_)^*T*^ be the associated weight vector of *Ω*_*i*_ with some conditions *ω*_*i*_ ∈ [0,1] and $\sum_{i=1}^{n}\omega_{i}=1$.

**Proof**. To prove this theorem, we use mathematical induction on *n*. For *n* = 2, we have

$$FFOWG\left(\Omega_{1},\Omega_{2}\right)=\Omega_{\sigma \left(1\right)}^{\omega_{1}}⊗\Omega_{\sigma \left(2\right)}^{\omega_{2}}$$

Breaking down the components $\Omega_{\sigma (1)}^{\omega_{1}}$ and $\Omega_{\sigma (2)}^{\omega_{2}}$ in view of Definition 6, we obtain:

$$\begin{array}{l} \Omega_{\sigma \left(1\right)}^{\omega_{1}}=\left(\;\mu_{\Omega_{\sigma \left(1\right)}}^{\omega_{1}},\sqrt[{3}]{1-{\left(1-\nu_{\Omega_{\sigma \left(1\right)}}^{3}\right)}^{\omega_{1}}}\right)\\ \Omega_{\sigma \left(2\right)}^{\omega_{2}}=\left(\;\mu_{\Omega_{\sigma \left(2\right)}}^{\omega_{2}},\sqrt[{3}]{1-{\left(1-\nu_{\Omega_{\sigma \left(2\right)}}^{3}\right)}^{\omega_{2}}}\right) \end{array}$$

Then,

$$\begin{array}{ll} \Omega_{\sigma \left(1\right)}^{\omega_{1}}⊗\Omega_{\sigma \left(2\right)}^{\omega_{2}} & =\left(\;\mu_{\Omega_{\sigma \left(1\right)}}^{\omega_{1}},\sqrt[{3}]{1-{\left(1-\nu_{\Omega_{\sigma \left(1\right)}}^{3}\right)}^{\omega_{1}}}\right)⊗\left(\;\mu_{\Omega_{\sigma \left(2\right)}}^{\omega_{2}},\sqrt[{3}]{1-{\left(1-\nu_{\Omega_{\sigma \left(2\right)}}^{3}\right)}^{\omega_{2}}}\right)\\ & =\left(\mu_{\Omega_{\sigma \left(1\right)}}^{\omega_{1}}\mu_{\Omega_{\sigma \left(2\right)}}^{\omega_{2}},\sqrt[{3}]{1-{\left(1-\nu_{\Omega_{\sigma \left(1\right)}}^{3}\right)}^{\omega_{1}}{\left(1-\nu_{\Omega_{\sigma \left(2\right)}}^{3}\right)}^{\omega_{2}}}\right) \end{array}$$

Consequently,

$$FFOWG\left(\Omega_{1},\Omega_{2}\right)=\left(\prod_{i=1}^{2}\mu_{\Omega_{\sigma \left(i\right)}}^{\omega_{i}},\;\sqrt[{3}]{1-\prod_{i=1}^{2}{\left(1-\nu_{\Omega_{\sigma \left(i\right)}}^{3}\right)}^{\omega_{i}}}\right)$$

This means that the theorem works for *n* = 2.

Then, assuming the theorem is valid for *n* = *k* > 2, we obtain:

$$FFOWG\left(\Omega_{1},\Omega_{2},\ldots ,\Omega_{k}\right)={⊗}_{i=1}^{k}\Omega_{\sigma \left(i\right)}^{\omega_{i}}$$

$$FFOWG\left(\Omega_{1},\Omega_{2},\ldots ,\Omega_{k}\right)=\left(\prod_{i=1}^{k}\mu_{\Omega_{\sigma \left(i\right)}}^{\omega_{i}},\;\sqrt[{3}]{1-\prod_{i=1}^{k}{\left(1-\nu_{\Omega_{\sigma \left(i\right)}}^{3}\right)}^{\omega_{i}}}\right)$$

Now, for the case *n* = *k* + 1, we can express it as:

$$\begin{array}{l} FFOWG\left(\Omega_{1},\Omega_{2},\ldots ,\Omega_{k},\Omega_{k+1}\right)={⊗}_{i=1}^{k}\Omega_{\sigma \left(i\right)}^{\omega_{i}}⊗\Omega_{\sigma \left(k+1\right)}^{\omega_{k+1}}\\ \;=\left(\prod_{i=1}^{k}\mu_{\Omega_{\sigma \left(i\right)}}^{\omega_{i}},\;\sqrt[{3}]{1-\prod_{i=1}^{k}{\left(1-\nu_{\Omega_{\sigma \left(i\right)}}^{3}\right)}^{\omega_{i}}}\right)\\ \;⊗\left(\mu_{\Omega_{\sigma \left(k+1\right)}}^{\omega_{k+1}},\;\sqrt[{3}]{1-{\left(1-\nu_{\Omega_{\sigma \left(k+1\right)}}^{3}\right)}^{\omega_{k+1}}}\right) \end{array}$$

This mean that

$$FFOWG\left(\Omega_{1},\Omega_{2},\ldots ,\Omega_{k+1}\right)=\left(\prod_{i=1}^{k+1}\mu_{\Omega_{\sigma \left(i\right)}}^{\omega_{i}},\;\sqrt[{3}]{1-\prod_{i=1}^{k+1}{\left(1-\nu_{\Omega_{\sigma \left(i\right)}}^{3}\right)}^{\omega_{i}}}\right)$$

This proves that the theorem remains valid when *n* equals *k* + 1. Thus, it can be deduced that the assertion holds true for every value of *n*.

The following example shows the application of Theorem 5.

**Example 2**. Let *Ω*_1_ = (0.7, 0.5), *Ω*_2_ = (0.9, 0.5), *Ω*_3_ = (0.6, 0.8) and *Ω*_4_ = (0.8, 0.7) be four FFNs, and *ω* = (0.1, 0.2, 0.3, 0.4)^*T*^ be the associated weight vector of *Ω*_i_, where *i* = 1,2,3,4. Now we calculate the scores of *Ω*_*i*_, by means of Definition 4

$$S\left(\Omega_{1}\right)=0.218,S\left(\Omega_{2}\right)=0.604,S\left(\Omega_{3}\right)=-0.296,S\left(\Omega_{4}\right)=0.169$$

Since $S\left(\Omega_{2}\right)≻S\left(\Omega_{1}\right)≻S\left(\Omega_{4}\right)≻S\left(\Omega_{3}\right)$, then the permutation vector *Ω*_*σ*(*i*)_, is described as follows:

$$\left(\Omega_{\sigma \left(1\right)},\Omega_{\sigma \left(2\right)},\Omega_{\sigma \left(3\right)},\Omega_{\sigma \left(4\right)}\right)=\left(\left(0.9,0.5\right),\left(0.7,0.5\right),\left(0.8,0.7\right),\left(0.6,0.8\right)\right)$$

Consequently, in view of Definition 6, we obtain the following outcome:

$$FFOWG\left(\Omega_{1},\Omega_{2},\Omega_{3},\Omega_{4}\right)=\left(0.702,0.714\right)$$

**Theorem 6**. Let *i* = 1,2, …, *n* and $\Omega_{i}=\left(\mu_{\Omega_{i}},\;\nu_{\Omega_{i}}\right)$ be FFNs. Suppose that *ω* = (*ω*_1_, *ω*_2_, …, *ω*_*n*_)^*T*^ be the associated weight vector of *Ω*_*i*_ with some conditions *ω*_*i*_ ∈ [0,1] and $\sum_{i=1}^{n}\omega_{i}=1$. If *Ω*_*σ*(*i*)_ = *Ω*_*σ*(*j*)_ are mathematically identical where $\Omega_{\sigma (j)}=\left(\mu_{\Omega_{\sigma (j)}},\nu_{\Omega_{\sigma (j)}}\right)$ then,

$$FFOWG\left(\Omega_{1},\Omega_{2},\ldots ,\Omega_{n}\right)=\Omega_{\sigma \left(j\right)}$$

**Proof**. Given that *Ω*_*σ*(*i*)_ = *Ω*_*σ*(*j*)_ for all *i* and for some fixed *j* implying $\mu_{\Omega_{\sigma (i)}}=\mu_{\Omega_{\sigma (j)}}$ and $\nu_{\Omega_{\sigma (i)}}=\nu_{\Omega_{\sigma (j)}}$. Then by using the above fact in Definition 10, we get

$$\begin{array}{c} FFOWG\left(\Omega_{1},\Omega_{2},\ldots ,\Omega_{n}\right)=\left(\prod_{i=1}^{n}\mu_{\Omega_{\sigma \left(i\right)}}^{\omega_{i}},\;\sqrt[{3}]{1-\prod_{i=1}^{n}{\left(1-\nu_{\Omega_{\sigma \left(i\right)}}^{3}\right)}^{\omega_{i}}}\right)\\ =\left(\mu_{\Omega_{\sigma \left(j\right)}}^{\sum_{i=1}^{n}\omega_{i}},\sqrt[{3}]{1-{\left(1-\nu_{\Omega_{\sigma \left(j\right)}}^{3}\right)}^{\sum_{i=1}^{n}\omega_{i}}}\right)\\ =\left(\mu_{\Omega_{\sigma \left(j\right)}},\sqrt[{3}]{1-\left(1-\nu_{\Omega_{\sigma \left(j\right)}}^{3}\right)}\right)=\left(\mu_{\Omega_{\sigma \left(j\right)}},\sqrt[{3}]{\nu_{\Omega_{\sigma \left(j\right)}}^{3}}\right)=\left(\mu_{\Omega_{\sigma \left(j\right)}},\nu_{\Omega_{\sigma \left(j\right)}}\right) \end{array}$$

Consequently,

$$FFOWG\left(\Omega_{1},\Omega_{2},\ldots ,\Omega_{n}\right)=\Omega_{\sigma \left(j\right)}$$

**Theorem 7**. Let $\Omega^{-}=\left({\text{min}}_{i}\left\{\mu_{\Omega_{\sigma (i)}}\right\},{\text{max}}_{i}\left\{\nu_{\Omega_{\sigma (i)}}\right\}\right)$ and $\Omega^{+}=\left({\text{max}}_{i}\left\{\mu_{\Omega_{\sigma (i)}}\right\},{\text{min}}_{i}\left\{\nu_{\Omega_{\sigma (i)}}\right\}\right)$ are respectively the lower and upper bounds of the FFNs $\Omega_{i}=\left(\mu_{\Omega_{i}},\;\nu_{\Omega_{i}}\right)$ and *ω* = (*ω*_1_, *ω*_2_, …, *ω*_*n*_)^*T*^ be the associated weight vector of *Ω*_*i*_ with some conditions *ω*_*i*_ ∈ [0,1] and $\sum_{i=1}^{n}\omega_{i}=1$. Then

$$\Omega^{-}\leq FFOWG\left(\Omega_{1},\;\Omega_{2},\ldots ,\;\Omega_{n}\right)\leq \Omega^{+}$$

**Proof**. The proof of this theorem follows the same method as Theorem 3.

**Theorem 8**. Consider two collections of FFNs, represented as $\Omega_{i}=\left(\mu_{\Omega_{i}},\;\nu_{F_{i}}\right)$ and $\Omega_{i}^{\prime }=\left(\mu_{\Omega_{i}^{\prime }},\;\nu_{\Omega_{i}^{\prime }}\right)$, with *i* ranging from 1 to *n* and *ω* = (*ω*_1_, *ω*_2_, …, *ω*_*n*_)^*T*^ be the associated weight vector of *Ω*_*i*_ and $\Omega_{i}^{\prime }$ satisfying the constraints *ω*_*i*_ ∈ [0,1] and $\sum_{i=1}^{n}\omega_{i}=1$. If $\mu_{\Omega_{\sigma (i)}}\leq \mu_{\Omega_{\sigma \left(i\right)}^{\prime }}$ and $\nu_{\Omega_{\sigma (i)}}\geq \nu_{\Omega_{\sigma \left(i\right)}^{\prime }}$, then we can establish that:

$$FFOWG\left(\Omega_{1},\;\Omega_{2},\ldots ,\;\Omega_{n}\right)\leq FFOWG\left(\Omega_{1}^{\prime },\;\Omega_{2}^{\prime },\ldots ,\;\Omega_{n}^{\prime }\right)$$

**Proof**. The proof of this theorem follows the same method as Theorem 4.

## 4. Implementation of suggested Fermatean fuzzy ordered weighted aggregation operators in MADM problems

In this section, we developed a step-by-step mathematical mechanism to tackle MADM issues that involve FF information with the help of the proposed operators.

- Let *ϒ* = {*ϒ*_1_, *ϒ*_2_, …, *ϒ*_*m*_} be the set of alternatives.
- Consider a set of attributes *χ* = {*χ*_1_, *χ*_2_, …, *χ*_*n*_} corresponding to a weight vector *ω* = (*ω*_1_, *ω*_2_, …, *ω*_*n*_)^*T*^, where *ω*_*i*_ ≥ 0 for *i* = 1, 2, …, *n*, and $\sum_{i=1}^{n}\omega_{i}=1$.
- Let $R=[{f_{ji}]}_{m\;\times \;n}={\left(\mu_{ji},\;\nu_{ij}\right)}_{m\;\times n}$ represents the FF decision matrix, where *μ*_*ji*_ and *ν*_*ji*_ indicate the extents to which alternative *ϒ*_*j*_ fulfills and is unable to fulfill attribute *χ*_*i*_, respectively. The following values follow the given circumstances:
$$\mu_{ji}\in \left[0,1\right],\nu_{ji}\in \left[0,1\right]\;\text{and}\;{\left(\mu_{ji}\right)}^{3}+{\left(\nu_{ji}\right)}^{3}\leq 1$$
  Using the decision knowledge previously provided, we devised an efficient MADM method to choose and rank the best alternatives.

### 4.1. Process for FFOWA and FFOWG

*Step 1*. Obtain decision matrix $R=[{f_{ji}]}_{m\;\times \;n}$ in the form of FFNs for alternatives relative to attributes.

*Step 2*. In order to obtain the FF permuted decision matrix $R_{\sigma }=[{f_{\sigma (ji)}]}_{m\;\times \;n}=\left(\mu_{\sigma (ji)},\nu_{\sigma (ji)}\right)$, we adopt the following two stages:

- Obtain the score values of all criterion *χ*_*i*_, corresponding to each alternative *ϒ*_*j*_ by means of Definition 4.
- Obtain the FF permuted decision matrix by arranging the computed values from the above stage of all criterion *χ*_*i*_, corresponding to each alternative *ϒ*_*j*_ in descending order.

*Step 3*. Utilized the developed FFOWA operator to amalgamate all the preference values *f*_*j*_ = (*μ*_*j*_, *ν*_*j*_) of all *ϒ*_*j*_ as follows:

$$FFOWA\left(f_{\sigma \left(j1\right)},\;f_{\sigma \left(j2\right)},\ldots ,\;f_{\sigma \left(jn\right)}\right)=\left(\sqrt[{3}]{1-\prod_{i=1}^{n}{\left(1-\mu_{\sigma \left(ji\right)}^{3}\right)}^{\omega_{i}}},\;\prod_{i=1}^{n}\nu_{\sigma \left(ji\right)}^{\omega_{i}}\right)$$

Likewise, in the FFOWG framework, the amalgamated values *f*_*j*_ are computed as follows:

$$FFOWG\left(f_{\sigma \left(j1\right)},\;f_{\sigma \left(j2\right)},\ldots ,\;f_{\sigma \left(jn\right)}\right)=\left(\prod_{i=1}^{n}\mu_{\sigma \left(ji\right)}^{\omega_{i}},\sqrt[{3}]{1-\prod_{i=1}^{n}{\left(1-\nu_{\sigma \left(ji\right)}^{3}\right)}^{\omega_{i}}}\right)$$

*Step 4*. Calculate the score values of *f*_*j*_ for each alternative *ϒ*_*j*_ utilizing Definition 4. If the score values for some alternatives become equal, then use the accuracy function defined in Definition 4 to calculate the score values for these alternatives.

*Step 5*. Assess the set of alternatives *ϒ*_*j*_ and determine which ones are optimal by arranging them with the help of $S(f_{j})$.

## 5. An optimal approach to reduce road traffic accidents under FF settings

In this section, we implement the offered methods of this article in an efficient manner to achieve an ideal technique for reducing RTAs using FF knowledge.

### 5.1. Case study

Transportation is responsible for a considerable amount of preventable fatalities [48]. According to WHO, RTAs cause an estimated 1.3 million fatalities annually, with an additional 20 to 50 million enduring non-fatal injuries that frequently lead to permanent disability [49]. The prevalence of road traffic injuries is higher in emerging economies, namely in low- and middle-income nations, which contribute to 93% of fatalities [49]. Car accidents are the primary cause of mortality in children and teenagers between the ages of 2 and 19, as supported by research [50,51]. Annually, over 186,300 individuals under the age of 19 lose their lives in road traffic accidents globally. In developing nations, the daily toll reaches over 500 fatalities, along with tens of thousands of lifelong injuries [52]. As a consequence, low and middle-income nations experience a threefold increase in the incidence of fatalities arising from road traffic incidents among this particular demographic, in contrast to high-income nations [53]. This disparity is attributed to the escalating velocity and volume of vehicular traffic in urban regions of developing countries, which adversely affects pedestrian safety and health [54].

Traffic accidents happen when a vehicle makes contact with another item. These obstacles can be attributed to factors such as road obstructions, people, animals crossing or loitering, or stable impediments like trees or utility poles. Rear-end collisions, side impact collisions, rollovers, head-on collisions, sideswipe collisions, single-vehicle accidents, and multiple-vehicle pile-ups are among the most common forms of traffic accidents [55,56]. Road accidents have been identified as a significant contributor to global fatalities, as well as physical impairment. The WHO recently presented data that unequivocally demonstrates the fact. Reducing road accidents should be a priority for everyone, since it is a valid objective set by the European Union under the Decade of Action for Road Safety (2011–2020). The goal is to decrease the number of casualties in member countries by 50% by 2020. In accordance with internationally recognized standards, Portugal has successfully undertaken the task of positioning itself among the top 10 European nations with the most favorable accident rate. The resolution of the Ministers Council in May 2014, as part of the Mid-term review for 2013–2015, establishes a target for Road Safety in Portugal. This objective is to ultimately achieve zero fatalities and zero serious injuries, with a long-term perspective [57].

Traffic signal control is a mechnism designed to coordinate the timing of several traffic signals in a given region, with the objective of minimizing pauses and optimizing the flow of vehicles. The system performs control, surveillance, and maintenance operations. This includes regulating traffic flow by changing and synchronizing traffic signals at junctions, monitoring traffic conditions using vehicle detectors and overseeing equipment functionality by detecting any equipment faults. These functions enable a traffic management agency to meet traffic demand, exchange traffic information with other agencies, and administer and upkeep the traffic light control system. The complexity of traffic signal management ranges from basic systems that utilize historical data to establish fixed timing plans, to adaptive signal control, which optimizes timing plans for a network of lights based on real-time traffic circumstances [58]. Over the past several years, technology has seen continuous advancements, enhancing the quality of people’s lives. One example of this is traffic management. Traffic lights were first equipped with gas-based illumination, but it quickly transitioned into a completely electrical system. In modern times, traditional traffic lights utilize LED technology because of their little energy consumption [59].

The three colors that indicate the right of way assigned to users adhere to a universally recognized color code:

- Red: Prohibits the passage of vehicles and allows pedestrians to cross. It has a duration of 28 to 40 seconds.
- Amber: Indicates an imminent transition of the traffic signal from green to red. It has a duration of 2 to 5 seconds.
- Green: Permits the passage of vehicles and signals the prohibition of pedestrian crossing. It has a duration ranging from 28 to 40 seconds.

The relationship between safer roads and police enforcement is intimately linked, as the latter actively promotes better road user behavior by ensuring compliance with fundamental traffic regulations and laws. Gaining insight into the correlations among law enforcement, driving conduct, and road safety is a fundamental requirement for maximizing the effectiveness of enforcement tactics. The implementation of traffic enforcement is carried out by appropriate government agencies and is directed towards road users. Its objective is to uphold favorable traffic conduct through the methods of monitoring, prosecution, and penalization [60]. The traffic violation, which refers to an unlawful driving action, serves as a connection between police enforcement and accidents. Violations are impacted by police enforcement and also have the potential to result in crashes. Determining the most efficient adjustments, evaluating the quality of the public transit transport supply, and ensuring that employees have access to convenient transportation are all tasks that can be accomplished with the assistance of MADM problems [61,62].

In following discussion, we present step by step mechanism to choose an appropriate method to reduce RTAs by means of the proposed strategies under FF environment.

### 5.2. Illustration

The administration of certain city wants to implement measures to reduce road traffic accidents. The administration has identified five different alternatives {*ϒ*_1_, *ϒ*_2_, *ϒ*_3_, *ϒ*_4_, *ϒ*_5_}, for improving road safety, where

1. *ϒ*_1_: Speed limit reduction
2. *ϒ*_2_: Public awareness campaign
3. *ϒ*_3_: Traffic signal optimization
4. *ϒ*_4_: Law enforcement
5. *ϒ*_5_: Road maintenance

The decision-makers want to use MADM techniques to select the best option based on five attributes {*χ*_1_, *χ*_2_, *χ*_3_, *χ*_4_, *χ*_5_}, where

1. *χ*_1_: Maintenance requirements
2. *χ*_2_: Effectiveness in accident reduction
3. *χ*_3_: Public acceptance
4. *χ*_4_: Motorist education
5. *χ*_5_: Impact on traffic flow

The decision-maker will assess the five potential alternatives *ϒ*_*j*_ in accordance with the FF data and the attributes *χ*_*i*_. The attribute weight vector is denoted by *ω* = (0.05, 0.1, 0.15, 0.3, 0.4)^*T*^. It can be seen that $\sum_{i=1}^{5}\omega_{i}=1$.

Table 3 summarizes the decision-maker’s opinion on each alternative for each attribute in the form of FFN. Decision matrix R is as follows.

**Table 3:** FF decision matrix R.

	*χ*_1_	*χ*_2_	*χ*_3_	*χ*_4_	*χ*_5_
*ϒ*_1_	(0.8,0.3)	(0.7,0.6)	(0.6,0.4)	(0.9,0.6)	(0.8,0.5)
*ϒ*_2_	(0.6,0.3)	(0.7,0.5)	(0.6,0.9)	(0.9,0.3)	(0.7,0.8)
*ϒ*_3_	(0.9,0.4)	(0.8,0.5)	(0.9,0.3)	(0.7,0.4)	(0.6,0.5)
*ϒ*_4_	(0.8,0.7)	(0.7,0.6)	(0.9,0.5)	(0.8,0.3)	(0.7,0.4)
*ϒ*_5_	(0.9,0.5)	(0.8,0.4)	(0.7,0.6)	(0.6,0.4)	(0.9,0.5)

The given MADM problem is analyzed using the FFOWA and FFOWG operators. We provide the technique and outcomes of two separate approaches that are employed to address this intricate decision problem.

*Step 1*. The permuted FF decision matrix $R_{\sigma }=[{f_{\sigma (ji)}]}_{5\;\times \;5}=\left(\mu_{\sigma (ji)},\nu_{\sigma (ji)}\right)$, is determined as follows:

- Obtain the score values of all five criteria corresponding to each alternative by means of Definition 4 as follows:
  - For alternative *ϒ*_1_, we have
    $S\left(f_{11}\right)=0.485,\;S\left(f_{12}\right)=0.127,\;S\left(f_{13}\right)=0.152$, $S\left(f_{14}\right)=0.513$ and $S\left(f_{15}\right)=0.387$
  - For alternative *ϒ*_2_, we have
    $S\left(f_{21}\right)=0.189,\;S\left(f_{22}\right)=0.218,\;S\left(f_{23}\right)=-0.513$, $S\left(f_{24}\right)=0.702$ and $S\left(f_{15}\right)=-0.169$
  - For alternative *ϒ*_3_, we have
    $S\left(f_{31}\right)=0.665,\;S\left(f_{32}\right)=0.387,\;S\left(f_{33}\right)=0.702$, $S\left(f_{34}\right)=0.279$ and $S\left(f_{35}\right)=0.091$
  - For alternative *ϒ*_4_, we have
    $S\left(f_{41}\right)=0.169,\;S\left(f_{42}\right)=0.127,\;S\left(f_{43}\right)=0.604$, $S\left(f_{44}\right)=0.485$ and $S\left(f_{45}\right)=0.279$
  - For alternative *ϒ*_5_, we have
    $S\left(f_{51}\right)=0.604,\;S\left(f_{52}\right)=0.448,\;S\left(f_{53}\right)=0.127$, $S\left(f_{54}\right)=0.152$ and $S\left(f_{55}\right)=0.604$
- Arrange the obtained values from the above stage corresponding to each alternative in descending order as follows:
  - For alternative *ϒ*_1_, we have
    $S\left(f_{14}\right)≻S\left(f_{11}\right)≻S\left(f_{15}\right)≻S\left(f_{13}\right)≻S\left(f_{12}\right)$
  - For alternative *ϒ*_2_, we have
    $S\left(f_{24}\right)≻S\left(f_{22}\right)≻S\left(f_{21}\right)≻S\left(f_{25}\right)≻S\left(f_{23}\right)$
  - For alternative *ϒ*_3_, we have
    $S\left(f_{33}\right)≻S\left(f_{31}\right)≻S\left(f_{32}\right)≻S\left(f_{34}\right)≻S\left(f_{35}\right)$
  - For alternative *ϒ*_4_, we have
    $S\left(f_{43}\right)≻S\left(f_{44}\right)≻S\left(f_{45}\right)≻S\left(f_{41}\right)≻S\left(f_{42}\right)$
  - For alternative *ϒ*_5_, we have
    $S\left(f_{51}\right)≻S\left(f_{55}\right)≻S\left(f_{52}\right)≻S\left(f_{54}\right)≻S\left(f_{53}\right)$

*Step 2*. Formulate the permuted FF decision matrix $R_{\sigma }=[{f_{\sigma (ji)}]}_{5\;\times \;5}=\left(\mu_{\sigma (ji)},\nu_{\sigma (ji)}\right)$, in the framework of the information obtained from step 1 Table 4 represents this matrix.

**Table 4:** Permuted FF decision matrix $R_{\sigma }$.

	*χ*_1_	*χ*_2_	*χ*_3_	*χ*_4_	*χ*_5_
*ϒ*_1_	(0.9,0.6)	(0.8,0.3)	(0.8,0.5)	(0.6,0.4)	(0.7,0.6)
*ϒ*_2_	(0.9,0.3)	(0.7,0.5)	(0.6,0.3)	(0.7,0.8)	(0.6,0.9)
*ϒ*_3_	(0.9,0.3)	(0.9,0.4)	(0.8,0.5)	(0.7,0.4)	(0.6,0.5)
*ϒ*_4_	(0.9,0.5)	(0.8,0.3)	(0.7,0.4)	(0.8,0.7)	(0.7,0.6)
*ϒ*_5_	(0.9,0.5)	(0.9,0.5)	(0.8,0.4)	(0.6,0.4)	(0.7,0.6)

*Step 3*. Utilized the FFOWA operator to aggregate all the preference values *f*_*j*_ of each *ϒ*_*j*_ as given in Table 5.

**Table 5:** Aggregate assessments of alternatives using the FFOWA operator.

Alternatives	*f*_*j*_
*ϒ*_1_	(0.727,0.482)
*ϒ*_2_	(0.674,0.657)
*ϒ*_3_	(0.742,0.445)
*ϒ*_4_	(0.761,0.546)
*ϒ*_5_	(0.748,0.486)

Similarly, utilized the FFOWG operator to aggregate all the preference values *f*_*j*_ of each *ϒ*_*j*_ as given in Table 6.

**Table 6:** Aggregate assessments of alternatives using the FFOWG operator.

Alternatives	*f*_*j*_
*ϒ*_1_	(0.699,0.520)
*ϒ*_2_	(0.651,0.809)
*ϒ*_3_	(0.697,0.458)
*ϒ*_4_	(0.747,0.599)
*ϒ*_5_	(0.708,0.515)

*Step 4*. In order to rank all the alternatives *ϒ*_*j*_ in the framework of FFOWA, compute the scores $S\left(f_{j}\right)$ of the entire FF preferences values *f*_*j*_, where *j* = 1,2,3,4,5. This is accomplished by applying Definition 6 as follows:

$$\begin{array}{l} S\left(f_{1}\right)=0.272S\left(f_{2}\right)=0.022S\left(f_{3}\right)=0.320\\ S\left(f_{4}\right)=0.277S\left(f_{5}\right)=0.303 \end{array}$$

Similarly, in order to rank all the alternatives *ϒ*_*j*_ in the framework of FFOWG, compute the scores $S\left(f_{j}\right)$ of the entire FF preferences values *f*_*j*_. This is again accomplished by applying Definition 6 as follows:

$$\begin{array}{l} S\left(f_{1}\right)=0.200S\left(f_{2}\right)=-0.253S\left(f_{3}\right)=0.242\\ S\left(f_{4}\right)=0.201S\left(f_{5}\right)=0.218 \end{array}$$

*Step 5*. The ranking order of the alternatives within FFOWA and FFOWG framework is established, revealing that *ϒ*_3_ ≻ *ϒ*_5_ ≻ *ϒ*_4_ ≻ *ϒ*_1_ ≻ *ϒ*_2_. Hence Traffic signal optimization is optimal choice to reduce RTAs.

**Fig 1:**
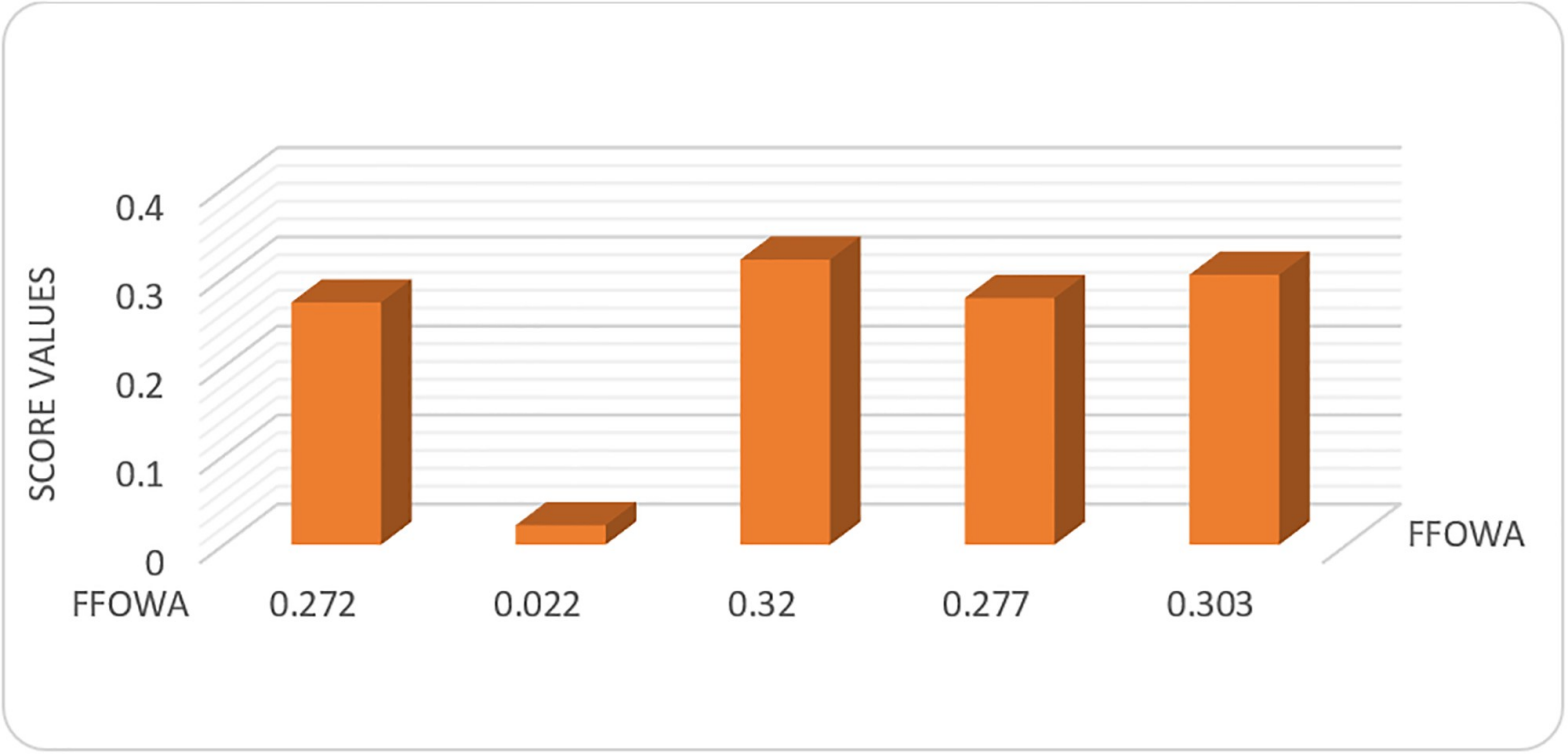
Ranking of alternatives using FFOWA.

**Fig 2:**
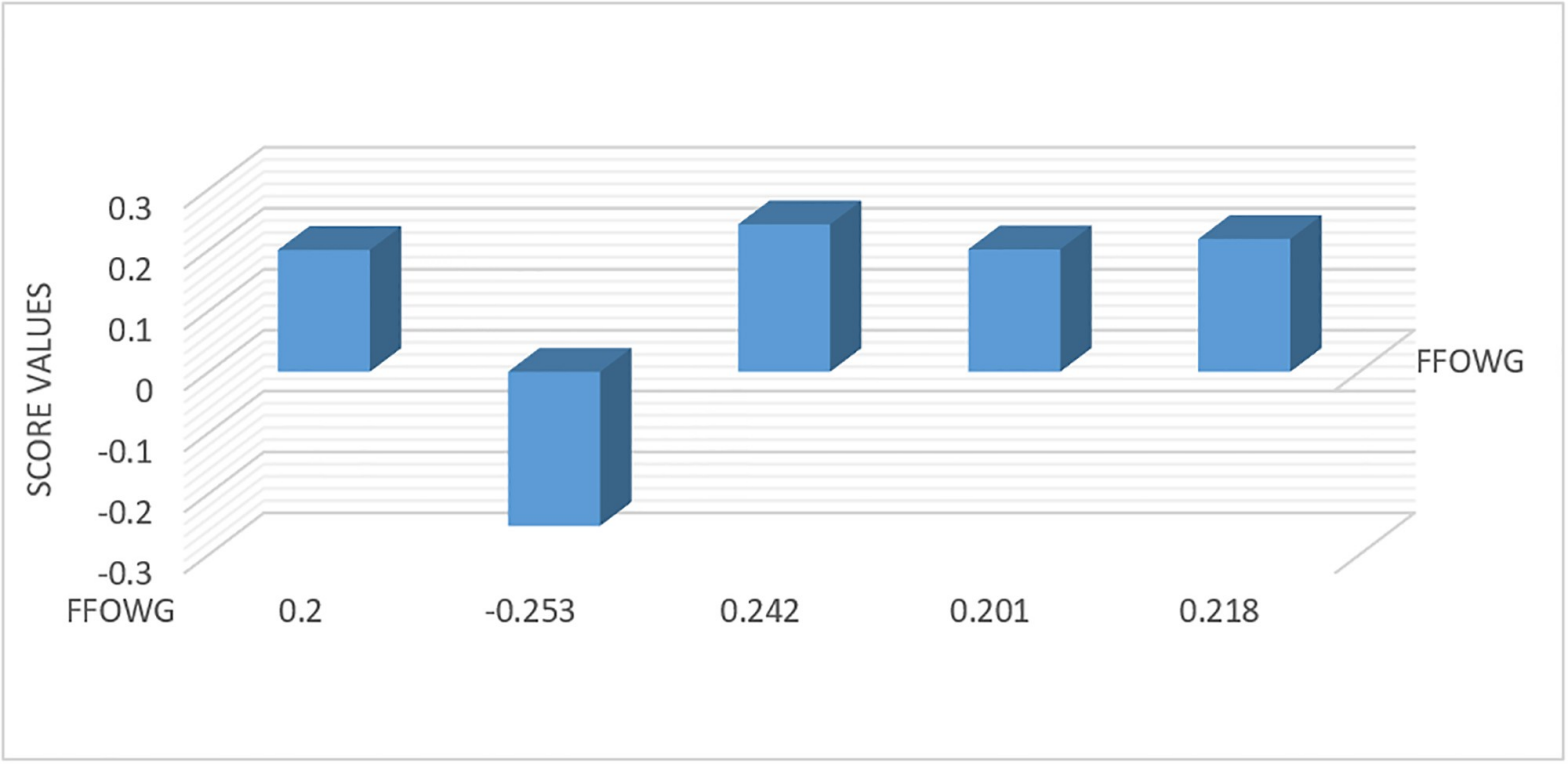
Ranking of alternatives using FFOWG.

The aforementioned procedure is graphically illustrated in Figs 1 and 2, which displays the score values of the alternatives obtained from the FFOWA and FFOWG operators respectively.

### 5.3. Comparative analysis

In the subsequent section, we address the aforementioned MADM problem by evaluating the effectiveness and credibility of our suggested operators in comparison to different operators in the IF, PF and FF environments. We employ many strategies, namely IFWA [3], IFWG [4], IFOWA [3], IFOWG [4], PFWA [16], PFWG [16], PFOWA [17], PFOWG [19], FFWA [22] and FFWG [22] operators, to collect and combine identical data. The outcomes obtained by utilizing these operators are consolidated in Table 7 and arranged in Table 8 based on their ranking.

**Table 7:** Aggregated values of the alternatives obtained from different existing operators.

	IFWA[3]	IFWG[4]	PFWA[16]	PFWG[16]	FFWA[22]
*f*(*ϒ*_1_)	(0.812,0.507)	(0.783,0.522)	(0.814,0.507)	(0.783,0.526)	(0.818,0.507)
*f*(*ϒ*_2_)	(0.771,0.551)	(0.731,0.693)	(0.776,0.551)	(0.731,0.716)	(0.781,0.551)
*f*(*ϒ*_3_)	(0.740,0.428)	(0.701,0.439)	(0.746,0.428)	(0.701,0.443)	(0.752,0.428)
*f*(*ϒ*_4_)	(0.779,0.406)	(0.761,0.432)	(0.781,0.406)	(0.761,0.443)	(0.783,0.406)
*f*(*ϒ*_5_)	(0.808,0.469)	(0.758,0.479)	(0.813,0.469)	(0.758,0.483)	(0.818,0.469)
	FFWG[22]	IFOWA [3]	IFOWG [4]	PFOWA [17]	PFOWG [19]
*f*(*ϒ*_1_)	(0.783,0.530)	(0.729,0.499)	(0.703,0.519)	(0.733,0.499)	(0.703,0.525)
*f*(*ϒ*_2_)	(0.731,0.734)	(0.672,0.621)	(0.656,0.790)	(0.675,0.674)	(0.656,0.801)
*f*(*ϒ*_3_)	(0.701,0.447)	(0.731,0.445)	(0.697,0.453)	(0.736,0.445)	(0.697,0.455)
*f*(*ϒ*_4_)	(0.761,0.456)	(0.766,0.560)	(0.752,0.589)	(0.760,0.546)	(0.747,0.591)
*f*(*ϒ*_5_)	(0.758,0.487)	(0.739,0.486)	(0.708,0.503)	(0.736,0.467)	(0.697,0.488)

**Table 8:** Score values and ranking of alternatives under existing and newly proposed strategies.

Methods	$S\left(f_{1}\right)$	$S\left(f_{2}\right)$	$S\left(f_{3}\right)$	$S\left(f_{4}\right)$	$S\left(f_{5}\right)$	Ranking Order
IFWA [3]	0.305	0.220	0.312	0.373	0.339	*ϒ*_4_ ≻ *ϒ*_5_ ≻ *ϒ*_3_ ≻ *ϒ*_1_ ≻ *ϒ*_2_
IFWG [4]	0.261	0.038	0.262	0.329	0.279	*ϒ*_4_ ≻ *ϒ*_5_ ≻ *ϒ*_3_ ≻ *ϒ*_1_ ≻ *ϒ*_2_
PFWA [16]	0.405	0.298	0.373	0.445	0.441	*ϒ*_4_ ≻ *ϒ*_5_ ≻ *ϒ*_1_ ≻ *ϒ*_3_ ≻ *ϒ*_2_
PFWG [16]	0.336	0.021	0.295	0.382	0.341	*ϒ*_4_ ≻ *ϒ*_5_ ≻ *ϒ*_1_ ≻ *ϒ*_3_ ≻ *ϒ*_2_
FFWA [22]	0.417	0.309	0.346	0.413	0.444	*ϒ*_5_ ≻ *ϒ*_1_ ≻ *ϒ*_4_ ≻ *ϒ*_3_ ≻ *ϒ*_2_
FFWG [22]	0.331	−0.004	0.255	0.345	0.320	*ϒ*_4_ ≻ *ϒ*_1_ ≻ *ϒ*_5_ ≻ *ϒ*_3_ ≻ *ϒ*_2_
IFOWA [3]	0.230	0.051	0.286	0.206	0.253	*ϒ*_3_ ≻ *ϒ*_5_ ≻ *ϒ*_1_ ≻ *ϒ*_4_ ≻ *ϒ*_2_
IFOWG [4]	0.184	−0.134	0.244	0.163	0.205	*ϒ*_4_ ≻ *ϒ*_5_ ≻ *ϒ*_1_ ≻ *ϒ*_4_ ≻ *ϒ*_2_
PFOWA [17]	0.288	0.001	0.343	0.279	0.323	*ϒ*_3_ ≻ *ϒ*_5_ ≻ *ϒ*_1_ ≻ *ϒ*_4_ ≻ *ϒ*_2_
PFOWG [19]	0.218	−0.211	0.278	0.208	0.247	*ϒ*_3_ ≻ *ϒ*_5_ ≻ *ϒ*_1_ ≻ *ϒ*_4_ ≻ *ϒ*_2_
FFOWA	0.272	0.022	0.320	0.277	0.303	*ϒ*_3_ ≻ *ϒ*_5_ ≻ *ϒ*_4_ ≻ *ϒ*_1_ ≻ *ϒ*_2_
FFOWG	0.200	−0.253	0.242	0.201	0.218	*ϒ*_3_ ≻ *ϒ*_5_ ≻ *ϒ*_4_ ≻ *ϒ*_1_ ≻ *ϒ*_2_

It is evident from Table 8 that the optimal solution achieved through the implementation of the suggested operators remains unchanged when IFOWA [3], IFOWG [4], PFOWA [17], and PFOWG [19] operators are utilized. This demonstrates the validity of our proposed methods and their applicability to MADM problems.

Furthermore, it is evident that the IFWA [3], IFWG [4], IFOWA [3], and IFWOG [4] operators are capable of efficiently managing intuitionistic fuzzy data. PFWA [16], PFWG [17], PFOWA [18], and PFOWG [19] operators are similarly capable of effectively managing Pythagorean fuzzy information. Nonetheless, numerous decision-making scenarios demand FF data. Our research expands the flexibility with which decision-makers can apply FF data to their particular circumstances. Therefore, upon evaluating all factors, it becomes evident that the proposed operators offer decision-makers more dependable and efficient support.

### 5.4. Advantages

The primary advantage of our suggested methods is that the FFS possesses a broader structure compared to the IFS and PFS, as it fulfills the criterion *μ*^3^ *+ ν*^3^ *≤* 1. Thus, it is better suited for addressing decision-making situations that involve ambiguity. Moreover, it is apparent that the techniques described in references [3,4,17,19] represent a particular case of the novel strategies presented in this study.

### 5.5. Limitations

1. FFSs are unable to handle scenarios where the sum of the cubes of membership and non-membership values exceeds 1.
2. FFSs cannot handle model cases involving picture fuzzy information and spherical fuzzy information due to their restriction to accepting only two parameters.

To address these constraints in future research:

- We will implement the recommended methodologies while operating in complex Fermatean fuzzy environments.
- We will explore the applicability of the recommended methodologies in q-rung fuzzy environments.
- We will investigate how the recommended methodologies can be adapted to handle picture fuzzy and spherical fuzzy information effectively.

## 6. Conclusions

The objective of this article is to propose innovative approaches to decision-making challenges in ordered-weighted Fermatean fuzzy environment. In addition to introducing two aggregation operators, FFOWA and FFOWG, we have analyzed their numerous features. Moreover, an inventive methodology has been implemented to tackle Fermatean fuzzy MADM challenges. Through the implementation of the FFOWA and FFOWG operators, this approach effectively handles decision-related information. We have provided a concrete illustration of how these recently established methods might be applied to choose the most effective way to minimize RTAs. To underscore the significance and dependence of these fresh techniques in contrast to existing approaches, a comparative analysis is conducted.

In the future, the suggested operators can be utilized in many different domains to streamline MADM, such as identifying the most favorable investment opportunities, determining suitable medical treatments, allocating energy resources, ranking projects, evaluating performance, prioritizing healthcare initiatives, utilizing big data analytics tools, and managing inventory. We aim to examine suggested strategies in the environments of interval-valued Fermatean fuzzy sets and bipolar fuzzy sets. We also tend to investigate the validity of ordered weighted aggregation operators on advanced structures such as quasirung fuzzy sets [35]. Additionally, we will explore dynamic ordered weighted aggregation operators in the FF environment.
