# Correction: A novel perspective on the selection of an effective approach to reduce road traffic accidents under Fermatean fuzzy settings

**DOI:** 10.1371/journal.pone.0305489

**Published:** 2024-06-10

**Authors:** The PLOS ONE Staff

There is an error in the Funding statement. The correct Funding statement is as follows: This project is supported by the Researchers Supporting Project Number (RSP2024R317) King Saud University, Riyadh, Saudi Arabia.

In the Abstract section, the words “decision” and “making” are written as single word. In all cases, “decisionmaking” should be “decision-making”.

The publisher apologizes for the errors.
